# Identification of novel monosodium urate crystal regulated mRNAs by transcript profiling of dissected murine air pouch membranes

**DOI:** 10.1186/ar2435

**Published:** 2008-06-03

**Authors:** Frank Pessler, Christian T Mayer, Sung Mun Jung, Ed M Behrens, Lie Dai, Joseph P Menetski, H Ralph Schumacher

**Affiliations:** 1 Klinik und Poliklinik für Kinder und Jugendmedizin, Technische Universität Dresden, Fetscherstraße, 01307 Dresden, Germany; 2 Division of Rheumatology, The Children's Hospital of Philadelphia, Civic Center Blvd, Philadelphia, Pennsylvania 19104, USA; 3 Institut für Medizinische Mikrobiologie, Immunologie und Hygiene, Technische Universität München, Trogerstraße, 81675 München, Germany; 4 Division of Rheumatology, University of Pennsylvania, Spruce St, Philadelphia, Pennsylvania 19104, USA; 5 Faculty of Oriental Medicine, Department of Herbal Pharmacology, Kyung Hee University College of Oriental Medicine, Hoekidong, Dongdaemoonku, Seoul 130-701, Korea; 6 Division of Rheumatology, Second Affiliated Hospital, Sun Yat-sen University, Yan Jiang West Road, Guangzhou 510120, PR China; 7 Merck Research Laboratories, E. Lincoln Avenue, PO Box 2000, Rahway, New Jersey 07065, USA; 8 Division of Rheumatology, Veteran Affairs Medical Center, University and Woodland Avenues, Philadelphia, Pennsylvania 19104, USA

## Abstract

**Introduction:**

The murine air pouch is a bursa-like space that resembles the human synovial membrane. Injection of monosodium urate (MSU) crystals into the pouch elicits an acute inflammatory response similar to human gout. We conducted the present study to identify mRNAs that were highly regulated by MSU crystals in the pouch membrane.

**Methods:**

Air pouch membranes were meticulously dissected away from the overlying skin. Gene expression differences between MSU crystal stimulated and control membranes were determined by oligonucleotide microarray analysis 9 hours after injection of MSU crystals or buffer only. Differential regulation of selected targets was validated by relative quantitative PCR in time course experiments with dissected air pouch membranes and murine peritoneal macrophages.

**Results:**

Eleven of the 12 most highly upregulated mRNAs were related to innate immunity and inflammation. They included mRNAs encoding histidine decarboxylase (the enzyme that synthesizes histamine), IL-6, the cell surface receptors PUMA-g and TREM-1, and the polypeptides Irg1 and PROK-2. IL-6 mRNA rose 108-fold 1 hour after crystal injection, coinciding with a surge in mRNAs encoding IL-1β, tumour necrosis factor-α and the immediate early transcription factor Egr-1. The other mRNAs rose up to 200-fold within the subsequent 3 to 8 hours. MSU crystals induced these mRNAs in a dose-dependent manner in cultured macrophages, with similar kinetics but lower fold changes. Among the downregulated mRNAs, quantitative PCR confirmed significant decreases in mRNAs encoding TREM-2 (an inhibitor of macrophage activation) and granzyme D (a constituent of natural killer and cytotoxic T cells) within 50 hours after crystal injection.

**Conclusion:**

This analysis identified several genes that were previously not implicated in MSU crystal inflammation. The marked rise of the upregulated mRNAs after the early surge in cytokine and Egr-1 mRNAs suggests that they may be part of a 'second wave' of factors that amplify or perpetuate inflammation. Transcript profiling of the isolated air pouch membrane promises to be a powerful tool for identifying genes that act at different stages of inflammation.

## Introduction

The murine air pouch is an easily accessible bursa-like space that can be produced *de novo *in the dorsal subcutaneous tissue [1]. Within several days of injecting a small volume of air (2 to 3 ml), a membrane of several layers of cells, which consist mostly of fibroblasts, mononuclear cells and small blood vessels, grows around this air-filled space [1]. This membrane resembles the synovial membrane histologically and has important properties of the synovial lining, such as hyaluronic acid synthesis [2] and expression of the Ia antigen [1]. Inflammatory substances or micro-organisms can be injected easily into the pouch, leading to different forms of inflammation depending on the agent used. For instance, inflammation caused by monosodium urate (MSU) [3] and calcium pyrophosphate crystals [4], carrageenan [4], joint prosthesis debris [5] and bacterial cell wall components [6] has been studied in this model.

Gene profiling of intact tissues is potentially hampered by the presence of adjacent noninflamed tissue, which increases the complexity of the tissue and introduces background 'noise'. Upon incision of the overlying dorsal skin, the air pouch membrane appears relatively loosely attached to the overlying subcutaneous tissue. We thus reasoned that it should be possible to dissect the membrane away from the overlying tissues and use this isolated membrane to study tissue-wide gene expression changes in an inflamed tissue of minimal complexity. Here, we report a dissection method leading to the isolation of the pouch membrane from the surrounding tissue. Microarray analysis of dissected inflamed and control membranes, coupled with validation of differential expression by relative quantitative polymerase chain reaction (qPCR), revealed a high yield of genes involved in innate immune responses and identified highly inducible mRNAs that were previously not implicated in crystal-induced inflammation.

## Materials and methods

### Air pouches

**Figure 1:**
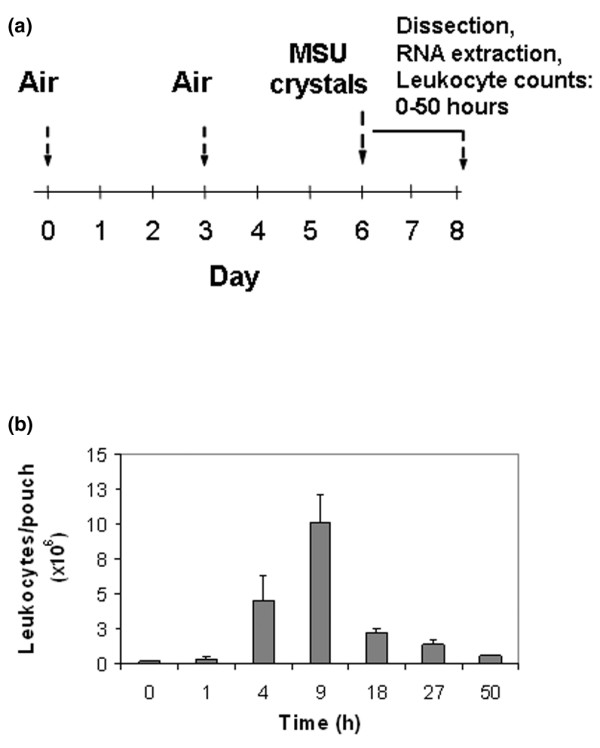
Outline of the air pouch experiments. **(a) **Sequence of events. Air is injected subcutaneously on day 0 and again on day 3 to keep the pouch inflated. On day 6 the remaining air is aspirated, and the monosodium urate (MSU) crystal suspension (2 mg in 1 ml phosphate-buffered saline [PBS]) or 1 ml PBS only is injected into the pouch cavity. Pouch exudate and tissue are obtained for analysis up to 50 hours after crystal injection. **(b) **Determination of the time of maximal inflammation in the pouch lumen. MSU crystal suspension (2 mg in 1 ml PBS) was injected into the pouch at t = 0 hours. Pouch exudate leukocyte counts were determined by manual cell counting at the indicated time points (*n* = 4 mice for each time point).

Figure 1a outlines the sequence of events of the air pouch experiments. Air pouches were raised on the backs of 6- to 8-week-old female BALB/c mice (Taconic, Tarrytown, NY, USA) by subcutaneous injection of 3 ml filtered air [1,7]. Pouches were re-inflated on day 3 with an additional 2 ml filtered air. MSU crystals were prepared in accordance with the method proposed by McCarty and Faires [8] and were determined to be free from endotoxin using the Gelclot LAL reagent (Charles River Labs, Wilmington, MA, USA). Aliquots from the same batch were used for all experiments. A suspension of 2 mg MSU crystals in 1 ml sterile endotoxin-free phosphate-buffered saline (PBS) was injected into the pouch on day 6. To verify the time points of peak and natural resolution of inflammation in the pouch, a 50-hour time course experiment was performed during which pouch exudate leukocyte counts were determined at several time points after injection of MSU crystals. In agreement with our previous findings [9], the leukocyte count rose 56-fold from 0 to 9 hours and then subsided, returning close to baseline by 50 hours (Figure 1b). Negative control pouches (*n* = 5) were injected with 1 ml PBS and harvested at 9 hours. Their mean leukocyte count was similar to that of the pouches at t = 0 hours (0.32 ± 0.18 × 10^6 ^cells/pouch at 9 hours versus 0.18 ± 0.09 at 0 hours; *p *= 0.31, one-tailed *t*-test). All animal experiments followed internationally recognized guidelines and were approved by the Institutional Animal Care and Research Committee of the Philadelphia VA Medical Center.

### Dissection of the air pouch membrane

**Figure 2:**
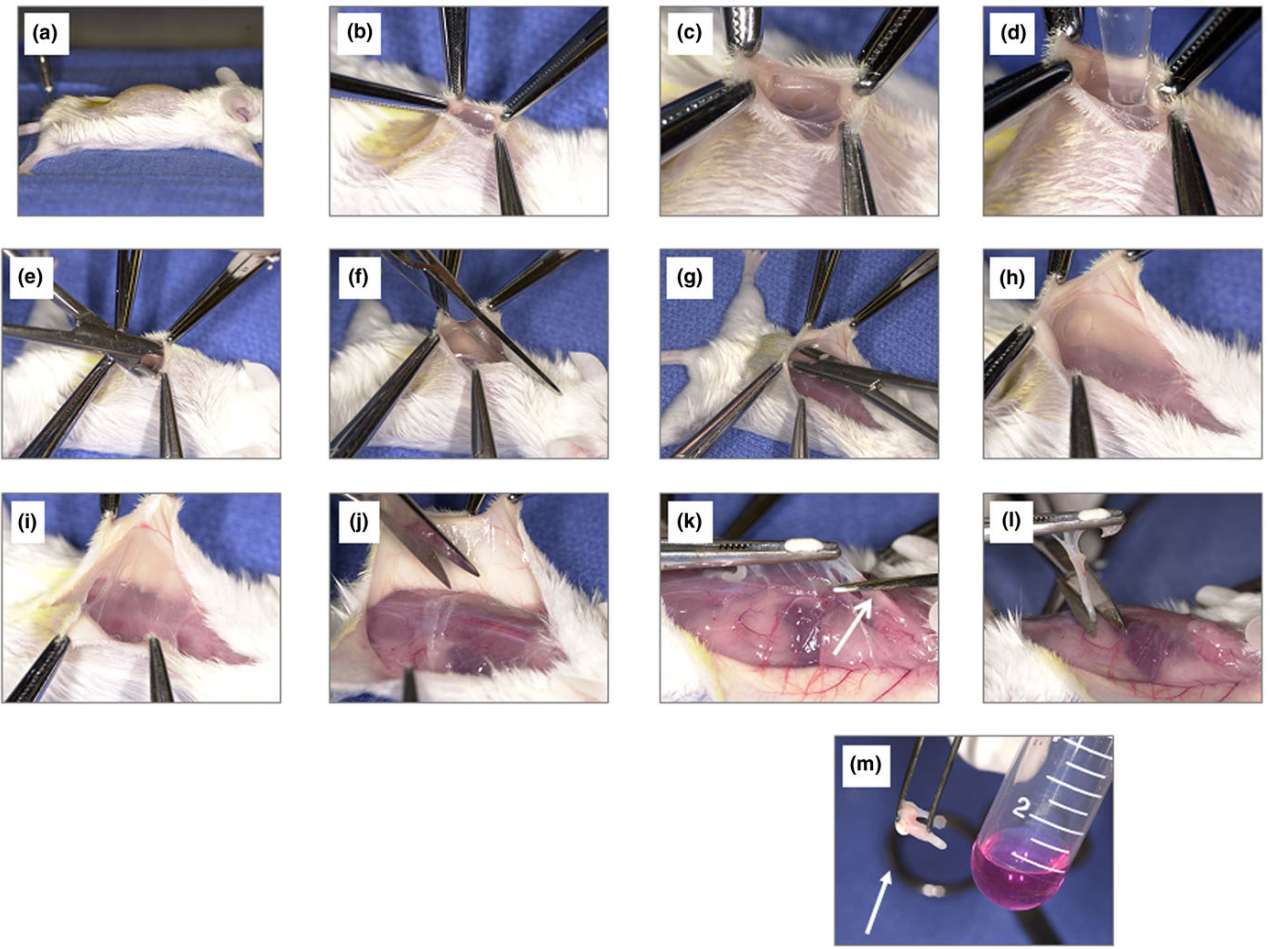
Key steps in the separation of the membrane from the overlying soft tissues. **(a) **Mouse with dorsal air pouch just before dissection. **(b) **A small incision is made into the dorsal skin overlying the pouch. This incision is just deep enough to expose the apex of the pouch membrane. **(c) **The membrane is punctured with a scalpel or needle. **(d) **The pouch content is lavaged with 2 ml phosphate-buffered saline. If the opening is enlarged sufficiently, the lavage can be performed under direct visualization. **(e-i) **Using blunt dissection with curved clamps or curved scissors, the pouch membrane is separated meticulously from the overlying skin. The instrument follows a path of least resistance between the membrane and the overlying soft tissues. **(j) **Parts of the membrane adhering to the more caudal skin can be clipped off with fine scissors. Finally, the membrane collapses on the dorsum of the animal. **(k, l) **The membrane is then grasped with forceps, elevated and cut at the base with scissors. **(m) **The gelatinous appearing dissected membrane (arrow) adhering to the forceps just before homogenization in Trizol medium.

Figure 2 shows key steps in the membrane dissection. After killing the animals by carbon dioxide asphyxiation, the apex of the pouch membrane was exposed with a small skin incision (panel b). The membrane was then punctured with a scalpel (panel c). Typically, little free exudate accumulates within the pouch. Leukocytes were therefore lavaged out of the pouch lumen with 2 ml PBS (panel d) and the leukocyte count in the resulting lavage fluid determined with a hemocytometer [10]. The pouch membrane was then separated meticulously from adjacent subcutaneous and paraspinal tissues by blunt dissection (panels e to j). Finally, the membrane was grasped with forceps, elevated and cut at the base (panels k and l). Great care was taken to avoid the paraspinal and nuchal tissues, to which the base of the membrane is typically attached. Using a rotatory homogenizer (Omni International, Warrenton, VA, USA), the isolated membranes were homogenized in Trizol medium (Invitrogen, Carlsbad, CA, USA), flash frozen in liquid nitrogen and stored at -70°C until further use.

### RNA extraction and gene expression analysis

RNA was extracted over RNeasy spin columns (Qiagen, Valencia, CA, USA) and tested for integrity and quantity on an Agilent 2100 Bioanalyzer (Agilent Technologies, Palo Alto, CA, USA). For unknown reasons, significant RNA degradation ensued when MSU-stimulated membranes, but not control membranes, were frozen and thawed before homogenization (data not shown). All membranes were therefore homogenized in Trizol medium immediately after dissection. Typically, 70 to 90 μg RNA was obtained per pouch.

For Affymetrix microarray analysis, RNA was processed and fluorescently labeled according to standard Affymetrix protocols [11]. To control for differences in dissection, RNA from three control or three MSU-stimulated pouch membranes was pooled, processed, labeled and then hybridized to Affymetrix Mo430_2 oligonucleotide microarrays (Affymetrix Inc., Santa Clara, CA). Microarray signals were scanned and analyzed using Affymetrix GCOS software and then imported into the software program GeneSpring version 7.2 (Silicon Genetics, Redwood City, CA, USA) for visualization and filtering. RNA aliquots were also reverse transcribed into cDNA according to standard protocols and analyzed further by qPCR using the TaqMan system and ABI Prism 7000 or 7900 HT sequence detectors (Applied Biosystems, Foster City, CA, USA). Commercially available primer-probe sets (Applied Biosystems) were used.

IL-6 protein concentration in the pouch exudate was determined by enzyme-linked immunosorbent assay (eBioscience, San Diego, CA, USA), after removal of cells and debris by centrifugation.

### Macrophages

Mouse peritoneal macrophages were harvested by peritoneal lavage 4 days after intraperitoneal injection of 2 ml aged 3% Brewer's thyoglycollate (Invitrogen Corporation, Carlsbad, CA, USA). Macrophages were allowed to rest in 3 ml tissue culture wells for 1 hour at 37°C (1 × 10^6 ^cells/well). After removal of nonadherent cells, cells were grown in medium with or without MSU crystals for the time periods indicated in the figure legends. Previously frozen cells were used for the dose response experiment. Macrophage cultures were more than 95% pure, as verified by flow cytometry for CD11b, major histocompatibility complex class II, and F4/80.

### Histology and immunohistochemistry

Tissues were obtained 9 hours after injecting MSU crystals into the pouch, fixed in formalin for 24 to 48 hours, and embedded in paraffin blocks. Blocks were cut into 5 μm thin sections on a rotatory microtome. Immunoperoxidase staining for IL-6 was performed using a semi-automated immunostaining system (DAKO, Carpinteria, CA, USA) and commercially available polyclonal goat anti-mouse IL-6 IgG (Santa Cruz Biotechnology Inc., Santa Cruz, CA, USA) at 1:100 dilution. Nonspecific goat IgG was used as negative control.

## Results

### Dissection of the pouch membrane

**Figure 3:**
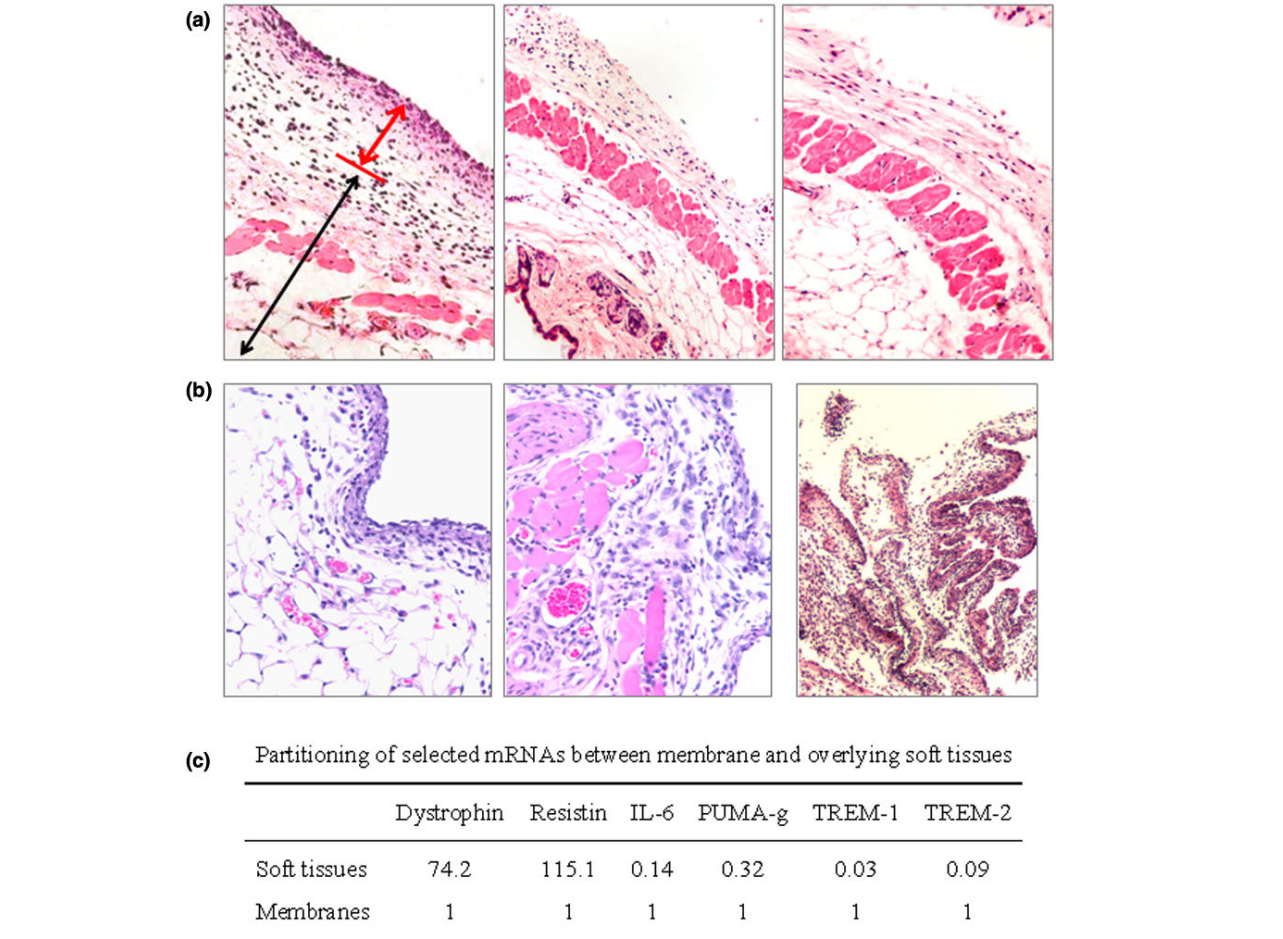
Histologic and molecular characterization of dissected membranes and adjacent tissues **(a) **Histologic cross-sections illustrating the plane of dissection. Left image: cross-section through an entire monosodium urate (MSU) crystal inflamed pouch wall, showing membrane (short red arrow) and the overlying cutaneous soft tissue (long black arrow). Original magnification: 100×. Center image: cutaneous soft tissue of the air pouch wall after removal of the membrane by the dissection method outlined in Figure 2. Original magnification: 100×. Right image: normal dorsal skin. It is nearly identical in appearance to the cutaneous parts shown in part b, which are left after dissection of the membrane. Original magnification: 100×. **(b) **Tissues that will probably contaminate the dissected membrane if they are not avoided during the final steps of the dissection. Left image: tissue from the nuchal cape-like structure to which the most rostral parts of the membrane are often attached. Original magnification: 200×. Center image: tissue obtained from the paraspinal ridges from which the base of the membrane arises (for the macroscopic appearance see the tissue marked with the arrow in Figure 2k). Original magnification: 200×. Right image: dissected membrane obtained from an air pouch injected with MSU crystals (2 mg in 1 ml phosphate-buffered saline). It consists mostly of fibroblasts and inflammatory cells. Blood vessels can also be found but are not abundant. Original magnification: 100×. **(c) **Partitioning of selected mRNAs between pouch membrane and the overlying cutaneous soft tissues. Membranes (*n* = 4) were dissected from the soft tissues 9 hours after injecting MSU crystals into the air pouches. RNA was extracted from dissected membranes or the soft tissues and analyzed separately by quantitative PCR. Results were normalized to GAPDH and the data obtained from membrane RNA arbitrarily assigned the value 1.

Crucial steps in the dissection procedure are shown in Figure 2 and are also described in the Materials and methods section (see above). Dissected pouch membranes had a gelatinous but also somewhat fibrous consistency and usually weighed 70 to 110 mg. Membranes from MSU-stimulated pouches tended to be firmer and to rupture somewhat less easily during the dissection, probably because of a mild increase in thickness from inflammation [12]. Figure 3a illustrates the plane of dissection between the membrane and the overlying subcutaneous tissue. According to our observations, the air pouch membrane originates from longitudinal soft tissue ridges that overlie the paraspinal musculature and from a cape-like, thicker membrane in the nuchal area. Because pieces of these tissues might contaminate the membrane during the dissection and confound a gene expression analysis, we evaluated them histologically (Figure 3b). The nuchal structure was identified as adipose tissue (Figure 3b, left image) and thus originates from the nuchal fat pad. The paraspinal ridge tissue (shown macroscopically in Fig. 2, panel k) turned out to be rich in blood vessels, striated muscle and fascia (Figure 3b, centre) and thus probably was contiguous with the paraspinal muscles. To test the histologic purity of the dissected membranes, haematoxylin and eosin stains were prepared from several membranes. Adipocytes, skeletal muscle and fascia were not observed, confirming that the membranes had been dissected relatively free from surrounding tissue (Figure 3b, right).

### Expression of selected targets in isolated membranes versus the overlying soft tissues

**Figure 4:**
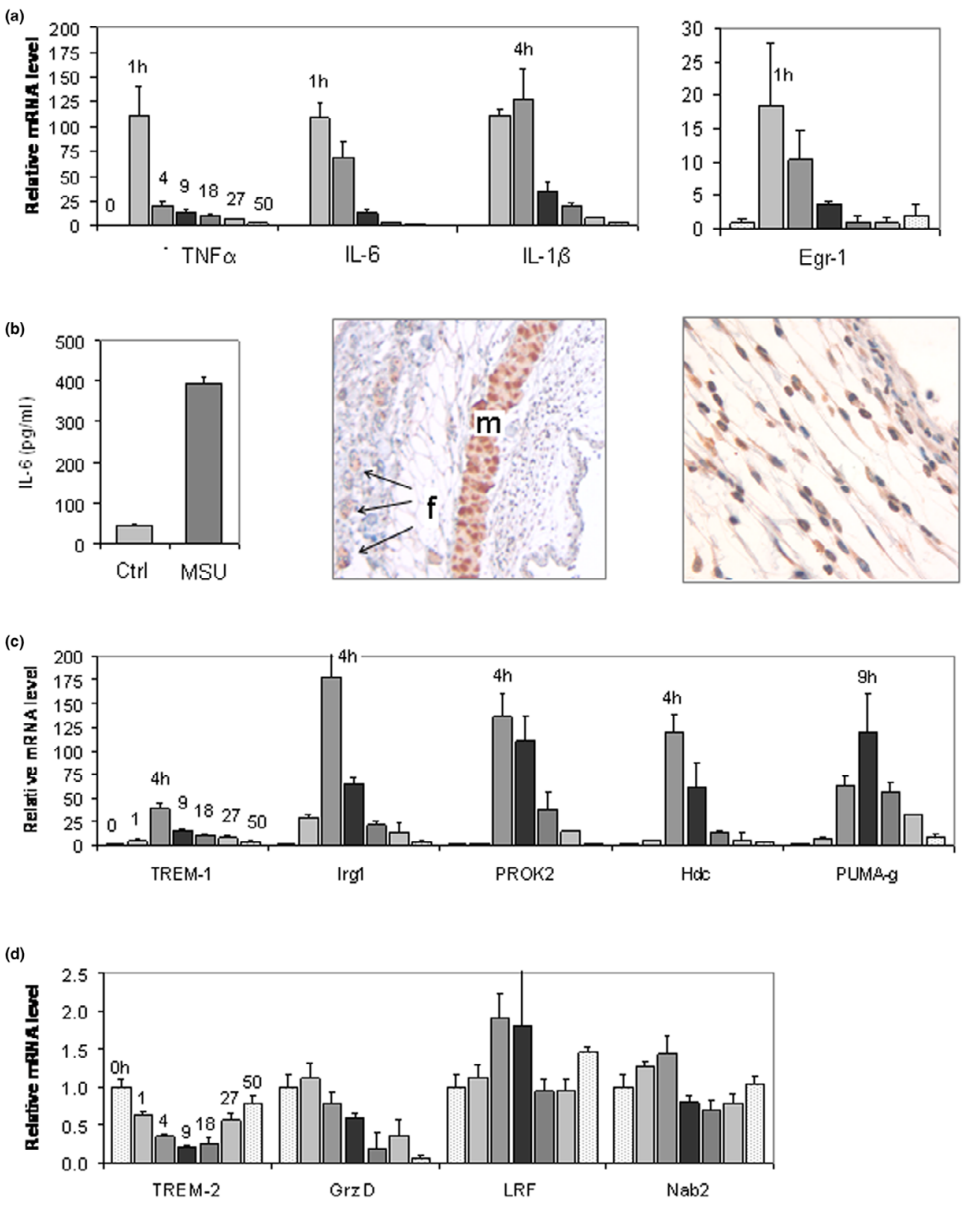
Validating differential regulation of mRNAs during MSU crystal inflammation in dissected air pouch membranes. Results were obtained from dissected membranes from the pouches used for the time course shown in Figure 1b. Negative control pouches were injected with 1 ml phosphate-buffered saline (PBS) and dissected at 9 hours. A, C and D: RNA was analyzed with TaqMan real-time reverse transcription PCR for the targets indicated. The legend text also shows values for mean fold expression changes that were measured at 9 hours (relative to 0 hours) in monosodium urate (MSU) crystal stimulated versus PBS injected (control) membranes. **(a) **mRNA quantification of tumour necrosis factor (TNF)-α (MSU:PBS at 9 h = 14.1:1.7), interleukin (IL)-6 (MSU:PBS = 12.9:0.5), IL-1β (MSU:PBS = 34.7:0.7), and early growth response (Egr)-1 (MSU:PBS = 3.7:1.4). **(b) **Left: determination of IL-6 protein concentration at 9 hours in the pouch exudate from pouches injected with PBS or MSU crystals (immunoassay). Center and right: immunohistochemical detection of IL-6 in the air pouch membrane. Chromogen: DAB (brown). Center: striated muscle (m) showing specific IL-6 immunostain; hair follicles (f) with nonspecific immunostain that was also seen with control immunoglobulin (original magnification, 50×). Right: specific IL-6 immunostaining in the inflamed pouch membrane (original magnification, 400×). **(c) **mRNA quantification of triggering receptor expressed on myeloid cells (TREM)-1 (MSU:PBS at 9 h = 15.3:0.6), immunoresponsive gene (Irg)1 (MSU:PBS, 65:1.0), prokineticin (PROK)-2 (MSU:PBS = 58.4:1.0), histidine decarboxylase (Hdc; MSU:PBS = 60.4:1.3), and protein upregulated on macrophages activated with interferon-γ (PUMA-g; MSU:PBS = 120:1.3). **(d) **mRNA quantification of TREM-2 (MSU:PBS at 9 h = 0.2:0.9), granzyme D (MSU:PBS = 0.5:0.8), leukemia/lymphoma-related factor (LRF; MSU:PBS = 1.8:1.7), and Nab2 (MSU:PBS = 0.8:0.9).

As expected, levels of mRNAs encoding resistin (a marker for adipocytes) and dystrophin (skeletal muscle) were by far highest in RNA extracted from the soft tissues overlying the membranes (Figure 3c). In contrast, mRNAs of several mRNAs identified by the microarray analysis of dissected membranes as inducible by MSU crystals (see below) were highest in membrane RNA. Consistent with the observations that IL-6 is also expressed in striated muscle (for instance, Figure 4b) and PUMA-g (protein upregulated on macrophages activated with interferon-γ) in adipocytes [13], the relative soft tissue fractions of these two mRNAs were larger than those of TREM (triggering receptor expressed on myeloid cells)-1 and TREM-2, both of which are predominantly expressed on inflammatory cells.

### Identification of differentially expressed genes by microarray analysis

Relative quantitative PCR (qPCR) analysis for resistin and dystrophin revealed that, despite the histologic absence of fat and skeletal muscle from the dissected membranes, individual noninflamed (control) membranes varied in the amounts of mRNAs encoding these markers. This suggested the presence of small remnants of fat and muscle on the membranes that persisted despite the histologically clean dissection. To correct for this heterogeneity and other potential differences resulting from the dissection, RNA aliquots from three control or three MSU-stimulated membranes (obtained 9 hours after injection of MSU crystals in PBS or PBS alone) were pooled. An aliquot from each pool was then processed according to standard Affymetrix protocols, and the resulting labelled cRNAs hybridized to separate Affymetrix Mo430_2 oligonucleotide microarrays. Of the 45,101 probe sets contained on the microarrays, 21,009 and 19,941 were detected (annotated 'p' in the raw Affymetrix data) in the pooled RNAs from control and MSU membranes, respectively. A total of 5,988 were differentially regulated (Affymetrix flag) in response to MSU crystals. These were filtered on the Affymetrix change *P *values below 2 × 10^-5 ^for upregulated and 1 for the downregulated probe sets.

Of these, the 12 most highly over-expressed (linear ratio MSU/control >17) targets are listed in Table 1. Eight (75%) were genes with known proinflammatory functions: histidine decarboxylase (Hdc, the enzyme that catalyzes the conversion of histidine to histamine); the surface receptors PUMA-g (also known as GPR109 or HM74) [14] and TREM-1 [15], which are induced on activated macrophages and neutrophils; immunoresponsive gene (Irg)1, a protein that is rapidly induced during monocyte activation with endotoxin [16]; prokineticin (PROK)-2, which is a small polypeptide that is involved in macrophage activation, hyperesthesia and other processes [14]; IL-6; colony-stimulating factor (CSF)3 (also known as granulocyte colony-stimulating factor); and the adhesion molecule P-selectin. Three genes (25%) encoded less well characterized factors but had potential functions in inflammation. One gene was uncharacterized. Of note, all of the over-expressed genes with proven proinflammatory functions related to innate immunity. Of the 12 most downregulated genes, three (25%) were related to connective tissue constituents, one (granzyme D) to innate immunity, and the others to various processes. The raw microarray data have been deposited in the Gene Express Omnibus microarray repository and are publically available under accession number GSE11498.

**Table 1:** The 12 air pouch membrane mRNAs most highly upregulated by MSU crystals

Gene	Genbank accession number	Ref.	Linear ratio: MSU/control	Function		Function(s)
					
				Inflammation^a^	MSU^b^	
PUMA-g (GPR 109/HM74)	NM 030701	[29]	64.2	+	-	Macrophage activation, prostaglandin synthesis
Hdc	AF 109137	[46]	33.8	+	±	Histamine synthesis
TREM-1	NM 021406	[15]	31.9	+	+	Expressed on activated macrophages, neutrophils
CSF3 (also known as G-CSF)	NM 007782	[47]	29.1	+	-	Granulocyte development and recruitment
Similar to MAPK phosphatase (cpg21; unpublished)	BB 442784	NA	27.9	±	-	Potentially, intracellular signal transduction, MAPK inactivation
IL-6	NM 031168	[48]	22.5	+	+	Multiple roles
PROK-2	NM 015768	[14]	21.0	+	-	Macrophage activation, hyperesthesia/pain, angiogenesis, gut motility
P-selectin	M 72332	[49]	20.1	+	-	Leucocyte, platelet adhesion to vascular endothelium
Similar to macrophage inflammatory protein 2 precursor	BB 829808	[50]	20.0	±	-	Putative neutrophil attracting chemokine
Irg1	L 38281	[16]	19.8	+	-	Induced in macrophages by LPS, mycobacteria
Dual specificity phosphatase 16	BB 121278	[51]	19.4	±	-	Potentially, intracellular signal transduction, MAPK inactivation
*Mus musculus *transcribed sequences (unpublished)	BB 993160	NA	18.0	-	-	Unknown

^a^Role of target gene in inflammation in general. ^b^Role of target gene in MSU crystal inflammation. +, known to play a role; ±, potentially involved; -, not known; CSF, colony-stimulating factor; G-CSF, granulocyte colony stimulating factor; Hdc, histidine decarboxylase; Irg, immunoresponsive gene; LRF, leukaemia/lymphoma-related factor; MAPK, mitogen-activated protein kinase; MSU, monosodium urate; NA, not applicable; PROK, prokineticin; PUMA-g, protein upregulated on macrophages activated with interferon-γ.

### Validation of IL-6 expression

IL-6 is known to be raised in gout [17], but it had not been studied in the MSU-stimulated murine air pouch. qPCR validation of its expression in the 50-hour time course experiment summarized in Figure 1 was therefore chosen to establish proof of principle of the microrray approach. Confirming the upregulation at 9 hours that had been detected with the microarrays (Table 1), IL-6 mRNA was 12.9-fold higher at this time point than at t = 0 hours (Figure 4a). However, peak induction occurred much earlier (1 hour) and was much higher (108-fold). In agreement with the observation that the pouch leukocyte count had returned close to its original level at the end of the 50-hour time course (Figure 1b), IL-6 mRNA also returned to near baseline by 50 hours. The mRNAs encoding IL-1β, tumour necrosis factor (TNF)-α, and the immediate early transcription factor early growth response (Egr)-1 [18] followed similar kinetics (Figure 4a). These findings confirmed induction of IL-6 mRNA in the membrane and identified the 1-hour time point as the peak of a rapid, early surge in proinflammatory cytokine and immediate early gene transcription.

In agreement with the induction of IL-6 mRNA in the membrane, analysis by enzyme-linked immunosorbent assay revealed that IL-6 protein levels at 9 hours were 8.7-fold higher in exudates from MSU-stimulated pouches than in exudates from control pouches (Figure 4b). The spatial expression of IL-6 in the inflamed air pouch was then determined immunohistochemically, using sections containing the pouch membrane adhering to the overlying skin. Consistent with previous reports of IL-6 expression in striated muscle [19], specific staining was seen in muscle fibres of the lamina muscularis of the subcutaneous tissue (Figure 4b, centre; marked 'm'), whereas the signal in hair follicles was not specific (marked 'f', arrows). In the inflamed membrane, specific IL-6 staining was seen in a multitude of cells, including mononuclear and polymorphonuclear cells and fibroblasts (Figure 4b, right).

### Validating induction of Hdc, TREM-1, PUMA-g, Irg1 and PROK-2 mRNAs by MSU crystals in the air pouch membrane

qPCR demonstrated dramatic induction of these mRNAs throughout the air pouch time course (Figure 4c). Confirming the microarray results, all of these mRNAs were elevated at 9 hours. Interestingly, the extent and kinetics of their induction differed; whereas maximal induction of Irg1 (177-fold), PROK-2 (136-fold), TREM-1 (39-fold) and Hdc (120-fold) occurred at 4 hours, PUMA-g mRNA was upregulated 120-fold and peaked at 9 hours.

To validate the results of the microarray analysis further, we determined kinetics of two mRNAs that were downregulated by MSU crystals, according to the array analysis (Figure 4d). TREM-2 is a homologue of TREM-1 that is downregulated during macrophage activation [20], and the microarray analysis had revealed an 81% decrease of its mRNA 9 hours after MSU crystal injection. Indeed, the time course demonstrated a 79% decrease in TREM-2 mRNA, with a nadir between 9 and 18 hours and subsequent recovery to near baseline level at 50 hours. In contrast, mRNA encoding granzyme D mRNA, which was 96% downregulated at 9 hours according to the microarrays, decreased steadily and reached 6% of its original level at 50 hours. As negative controls, we measured expression of two mRNAs that were not among the differentially regulated genes on the arrays (Figure 4d). First, leukaemia/lymphoma-related factor (LRF; also known as FBI-1 and OCZF), a transcription factor important in cellular transformation [21], osteoclastogenesis [22] and regulation of the HIV-1 promoter [23,24]. Levels of its mRNA demonstrated a statistically insignificant twofold increase at 4 hours. Second, mRNA encoding Nab2, a co-regulator of Egr-1 [25], exhibited only minor fluctuations. To exclude their origin from remnant adipose or muscle tissue, qPCR results for all of the above mRNAs at 9 hours were also correlated with resistin and dystrophin mRNA expression, but no significant associations were found.

### IL-6, Irg1, PROK-2, Hdc and PUMA-g mRNA upregulation in MSU-stimulated mouse peritoneal macrophages

**Figure 5:**
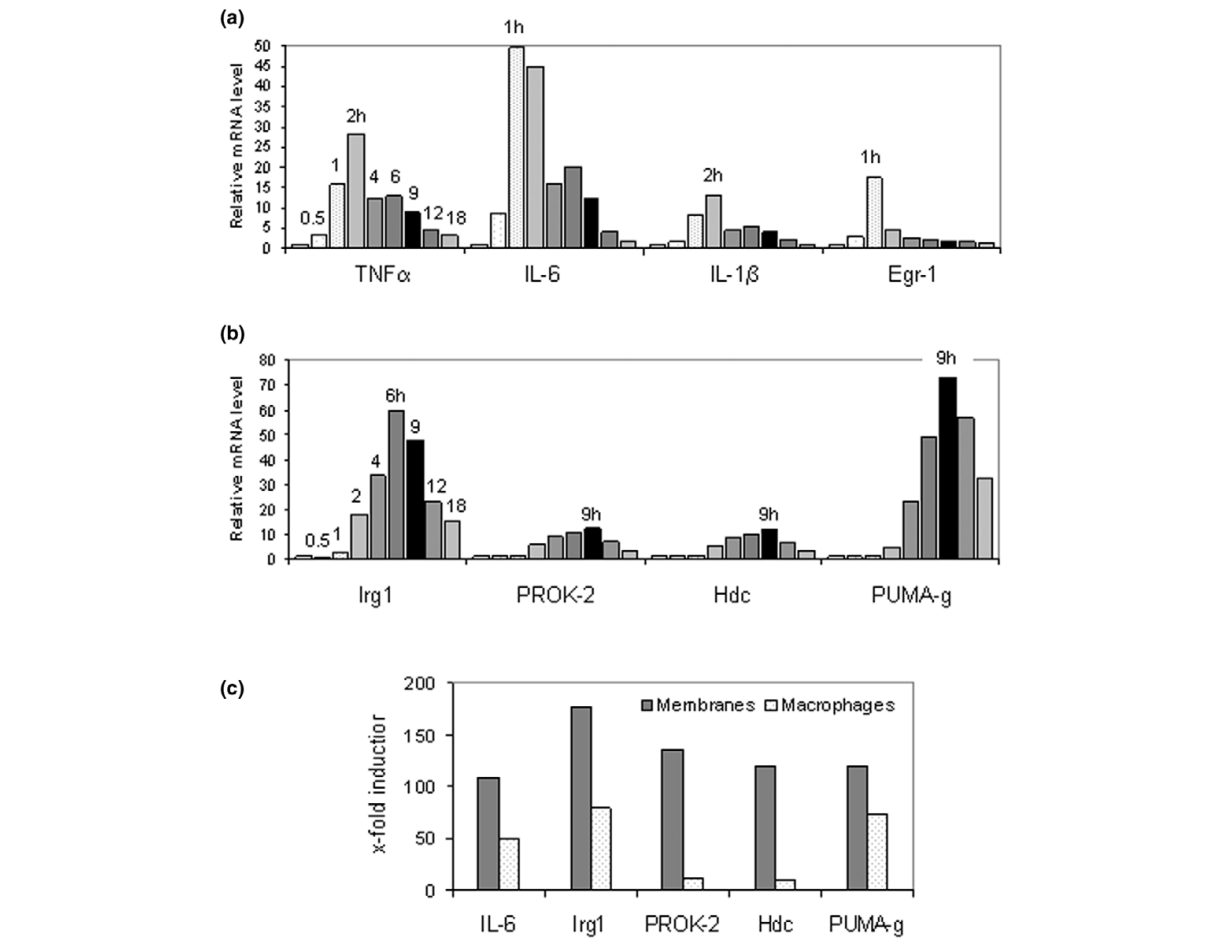
Validating differential regulation of mRNAs during MSU crystal inflammation in mouse peritoneal macrophages. Cells were harvested at the indicated time points after the addition of medium containing monosodium urate (MSU) crystals (200 μg/ml) or medium alone. RNA was analyzed by TaqMan real-time reverse transcription PCR for expression of the targets indicated in the figure. Results represent the averages of two experiments. Induction of target mRNAs in negative control (medium only) cells was negligible in nearly all cases. Therefore, the curves corresponding to the negative controls are not shown. However, numeric values for mean fold expression changes in MSU stimulated and negative controls with respect to t = 0 hours are listed below for the time points of maximal induction by MSU crystals. **(a) **Tumour necrosis factor (TNF)-α (2 hours: MSU:medium = 28.1:1.2), IL-6 (1 hour: MSU:medium = 49.7:1.2), IL-1β (2 hours: MSU:medium = 13.1:0.5); and early growth response (Egr)-1 (1 hour: MSU:medium = 17.4:1). **(b) **Irg1 (6 hours: MSU:medium = 60.0:6.8); prokineticin (PROK)-2 (6 hours: MSU:medium, 11.0:1.0), histidine decarboxylase (Hdc; 9 hours: MSU:medium = 11.6:0.9) and protein upregulated on macrophages activated with interferon-γ (PUMA-g; 9 hours: MSU:medium = 73.5:2.5). **(c) **Comparison of fold induction by MSU crystals in dissected membranes versus macrophage culture.

The induction of TREM-1 in macrophages by MSU crystals has been reported [26]. To test whether macrophages were also potential sources of Irg1, PROK-2, Hdc and PUMA-g, kinetics of these mRNAs were determined during 18 hours after the addition of MSU crystals to cultured mouse peritoneal macrophages (Figure 5). As in the tissue, MSU crystals induced sharp, early peaks in TNF-α, IL-6, IL-1β, and Egr-1 mRNAs (Figure 5a). MSU crystals also upregulated Irg1, PROK-2, Hdc, and PUMA-gmRNAs (Figure 5b). However, with the exception of PUMA-g, their levels peaked somewhat later than in the membranes. As illustrated in Figure 5c, these mRNAs were, to various extents, induced more highly in the membranes than in the macrophages. As in the membranes, MSU crystals caused a decrease in TREM-2 mRNA in the macrophages and no significant changes in Nab2 and LRF mRNAs (data not shown).

**Figure 6:**
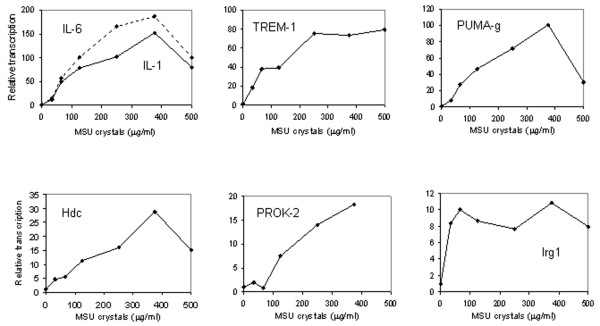
MSU crystal dose response. Mouse peritoneal macrophages were grown in medium overnight. After removal of nonadherent cells, medium containing increasing concentrations of monosodium urate (MSU) crystals was added. RNA was harvested 4 hours after the addition of crystal-containing medium and analyzed for target gene expression by TaqMan real-time reverse transcription PCR. Results represent the averages of two experiments.

The induction of IL-1β, IL-6, TREM-1, PUMA-g, Hdc, PROK-2 and Irg1 by MSU crystals was also quantified in a dose response experiment (Figure 6). IL-1β and IL-6 mRNAs were induced maximally at a crystal concentration of 375 μg/ml medium, as were PUMA-g, Hdc and PROK-2 mRNAs. Induction of TREM-1 mRNA peaked at a slightly lower, and that of Irg1 mRNA at the lowest crystal concentration.

## Discussion

Using microarray analysis of meticulously dissected air pouch membranes, we identified several genes relating to innate immunity that were induced strongly by MSU crystals. Four of the six factors whose upregulation by MSU crystals was confirmed by qPCR (PUMA-g, Irg1, Hdc and PROK-2) had not previously been associated with crystal-induced inflammation or inflammatory arthropathies. Since the initial presentation of our microarray results [27], the involvement of TREM-1 in MSU crystal-induced inflammation was reported independently [28], thus serving as an additional positive control and validating our approach.

Background signals from adjacent tissues potentially interfere with the use of genomic tools for the identification of genes that are expressed differentially in an inflamed tissue. In the case of the air pouch model, this interference would come from highly differentiated structures in the overlying subcutaneous and cutaneous tissues such as striated muscle, adipocytes, hair follicles, sweat glands and epithelium, all of which possess unique gene expression patterns that would complicate a microarray analysis further. In addition, they may express factors that might play important roles in inflammation when expressed in immune cells. Indeed, the immunostains revealed specific expression of IL-6 in the subcutaneous striated muscle, and PUMA-g is known to be expressed in adipocytes. As shown in Figure 3c, IL-6 and PUMA-g mRNAs were relatively abundant in the soft tissues overlying the membrane, whereas TREM-1 and TREM-2 mRNAs partitioned more preferentially to the membranes. If the microarray analysis had been performed with entire pouch walls, the baseline expression of IL-6 or PUMA-g mRNAs in myocytes and adipocytes might possibly have precluded a microarray-based identification of these mRNAs as being highly inducible by MSU crystals.

### Potential roles of PUMA-g, TREM-1, Irg1, PROK-2 and Hdc in the pathogenesis of crystal inflammation

We validated the induction of six of the most highly over-expressed genes by qPCR. How might these function in the pathogenesis of crystal-induced inflammation? PUMA-g is a G-protein-coupled transmembrane receptor that was initially identified in a microarray screen of macrophages activated with TNF-α and interferon-γ [29]. In addition to activated macrophages, it is also expressed on neutrophilic granulocytes and adipocytes. On the latter, it is a major receptor for the cholesterol-lowering agent nicotinic acid [13]. Binding of this ligand to PUMA-g is also responsible for the cutaneous flushing response that is frequently observed in patients taking nicotinic acid [30], and it has been proposed that cutaneously expressed PUMA-g leads to the release of prostaglandins D_2 _and E_2_, which in turn cause local vasodilatation [30]. These results suggest that in MSU crystal inflammation PUMA-g transmits signals in macrophages, and perhaps also granulocytes, which potentiate release of prostaglandins and thus enhance the inflammatory process. Further studies with cultured cells or PUMA-g^-/- ^mice will now be important in elucidating the function of PUMA-g in crystal-induced inflammation.

TREM-1, too, is induced on the surface of activated macrophages and neutrophils, and has been shown to play important roles in the systemic manifestations of sepsis [26]. Interestingly, Murakami and coworkers [28] showed that TREM-1 mRNA in the pouch exudate peaked before the maximum accumulation of leukocytes, thus indicating activation of TREM-1 transcription early on. It has been proposed that one function of TREM-1 is to amplify Toll-like receptor (TLR) signaling [26]. Considering that TREM-1 mRNA reached maximum levels after the initial cytokine mRNA surge (Figure 4a) and that recognition of MSU crystals via TLR signaling is believed to be among the earliest events in crystal inflammation [31], one may postulate that the role of TREM-1 in crystal inflammation also relates to potentiation of signals transmitted by TLRs.

Irg1 was isolated originally from a cDNA library made from endotoxin-stimulated macrophages [16], and was later found to be expressed in macrophages infected with mycobacteria [32,33]. Its induction by MSU crystals in peritoneal macrophages (Figures 5 and 6) suggests that it may play general roles in macrophage activation.

Prokineticins are evolutionary highly conserved small secreted polypeptides that play roles in various tissues, including the central nervous system, the gastrointestinal tract, the haematopoietic system and the vasculature [14,34,35]. PROK-2 was recently shown to augment macrophage chemotaxis and proinflammatory cytokine synthesis [36]. Interestingly, it also causes hyperesthesia, including by local injection into rodent paws, and mice lacking the PROK receptor PKR1 have diminished pain responses [37]. It is therefore intriguing to speculate that PROK-2 contributes to the symptoms of MSU crystal inflammation by enhancing both inflammation and pain in affected joints.

The enzyme Hdc converts histidine to histamine. Histamine is elevated in the joint fluid of patients with gout [38] and in the MSU crystal stimulated rat air pouch, where it is believed to be the result of mast cell degranulation [12]. Our results, however, suggest that histamine may also arise from increased Hdc levels during MSU crystal inflammation. Consistent with our observation that MSU crystals induced Hdc mRNA synthesis in macrophage culture, with reports of Hdc mRNA expression in neutrophils [39], and with the well documented presence of mast cells in the air pouch membrane [12], all of these three cell types are probably highly inducible sources of Hdc in the pouch membrane. Thus, Hdc and histamine may contribute significantly to the evolution of MSU crystal inflammation in this model, but also in gouty synovitis in humans.

### Significance of the downregulated genes

TREM-2 is a cell surface receptor similar to TREM-1. It promotes differentiation of macrophages into osteoclasts [40] but inhibits macrophage activation and is downregulated during this process [20]. The decrease in TREM-2 mRNA in both membranes and cultured macrophages suggests that macrophage activation by MSU crystals leads to an increase in TREM-1 but to a decrease in TREM-2 mRNA levels. Granzyme D has been detected in murine cytotoxic T cells [41] and endometrial natural killer (NK) cells [42]. The roles of these cell types in urate crystal inflammation have not been studied. However, it is known that uric acid can activate CD8^+ ^T cells under some circumstances [43], and in preliminary studies we have detected mRNA encoding the NK cell lectin-like receptor A2 in the air pouch membrane (Mayer CT, Schumacher HR, Pessler F, unpublished data). Thus, the persistent downregulation of granzyme D throughout the time course suggests that MSU crystals may modulate cytotoxic T cell or NK cell activity in the membrane. Alternatively, one must consider that dilution of the membrane RNA pool by mRNAs contained in immigrating inflammatory cells or by otherwise highly upregulated mRNAs might also cause – at least in part – artifactual decreases in mRNAs expressed by resident membrane cells.

### Kinetics of MSU crystal inflammation in the air pouch membrane

The results of the present study also shed new light on the kinetics of transcriptional regulation in the air pouch membrane. We observed an early, steep rise in proinflammatory cytokine mRNA synthesis that essentially paralleled the synthesis of mRNA encoding the immediate early transcription factor Egr-1. Similarly rapid kinetics were observed in cultured macrophages (Figure 5). Indeed, inclusion of the 30-minute time point revealed that increasing levels of mRNAs encoding Egr-1 and all three cytokines could be detected this early. It is currently believed that TLR-mediated activation of the Nalp3 containing inflammasome, leading to proteolytic cleavage of pre-existing pro-IL-1β to IL-1β, is among the first steps in MSU crystal inflammation [31,44]. Our findings suggest that activation of the proinflammatory cytokine genes encoding IL-1β, IL-6 and TNF-α, as well as Egr-1 and probably other immediate early transcription factors, is among the next, early events in reprogramming the transcriptional machinery in MSU crystal inflammation, both in an intact tissue and in cultured cells. TREM-1, Irg1, PROK-2, Hdc and PUMA-g mRNA levels all peaked after this dramatic burst in early cytokine transcription. These factors may therefore play roles in perpetuating or amplifying this early inflammation. We had performed the microarray analysis 9 hours after MSU crystal injection, the peak of leukocyte accumulation in the pouch lumen. However, results of the time course analysis demonstrate that major transcriptional reprogramming in the air pouch membrane occurs several hours earlier. Future RNA-based studies into events leading to the peak phase of inflammation in the membrane should therefore focus on relatively early time points up to 4 hours.

### Caveats of the present study

Gene expression results obtained from the dissected membranes do not allow one to determine whether a given overexpressed gene was induced in the resident membrane cells or imported by infiltrating cells. It is therefore important to validate differential expression of a given gene in a purified cell population, as was done here with cultured macrophages. Even though we validated the induction of all six targets in cultured macrophages, their fold induction was lower in these cells than in the membranes (Figure 5). This suggests that events in the membranes due to cell flux, expression in cells other than macrophages (for example, neutrophils), or more efficient gene regulation due to cell-cell interactions contribute significantly to regulation of these genes in the intact membranes. Another caveat is that the microarray analysis was not replicated, which precluded an extended statistical analysis. However, the unusually high degrees of over-expression and under-expression apparently compensated for this lack of replication; they allowed us to choose a small number of genes for qPCR validation strictly by the high magnitudes of their expression changes.

It is also important to remember that inflammation extends beyond the membrane into the loose, fibroblast-rich tissue between the membrane and the striated muscle layer (Figure 3a and Schiltz and coworkers [12]). The air pouch membrane therefore does not contain the entire inflammation that develops in the air pouch, and inflammatory cells may remain in the air pouch wall if the membrane is removed with the blunt dissection method described here. Furthermore, MSU crystals cause swelling of the air pouch membrane [45]. This is most likely due to the influx of inflammatory cells and associated interstitial edema and must be considered when evaluating the membrane histologically.

### Potential uses of the isolated air pouch membrane in gene discovery

Our findings suggest that a more extensive, replicated microarray analysis involving higher numbers of air pouch membranes may be a powerful tool to identify genes that are differentially active in various aspects of inflammation and innate immunity. In particular, it should lend itself well to the identification of genes that act at different time points in the evolution or resolution of inflammation, genes that are regulated in response to treatment with anti-inflammatory agents, or genes that reflect changes in the resident connective tissue cells in response to inflammation.

## Conclusion

A dissection method was established that allowed for the separation of the murine air pouch membrane from the overlying cutaneous tissues. Transcript profiling of this minimal tissue identified several mRNAs relating to innate immune responses that were previously not implicated in MSU crystal inflammation. The appearance of these mRNAs after an initial surge in proinflammatory and immediate early gene transcription suggests that they may form part of a 'second wave' of factors that amplify or perpetuate acute inflammation.

## Abbreviations

CSF = colony-stimulating factor; Egr = early growth response; Hdc = histidine decarboxylase; IL = interleukin; Irg = immunoresponsive gene; LRF = leukaemia/lymphoma-related factor; MSU = monosodium urate; NK = natural killer; PBS = phosphate-buffered saline; PCR = polymerase chain reaction; PROK = prokineticin; PUMA-g = protein upregulated on macrophages activated with interferon-γ; qPCR = quantitative polymerase chain reaction; TLR = Toll-like receptor; TNF = tumour necrosis factor; TREM = triggering receptor expressed on myeloid cells.

## Competing interests

The authors declare that they have no competing interests.

## Authors' contributions

FP designed the study, performed the experiments and wrote the manuscript. CTM, SMJ and LD participated in the experiments. EMB provided the macrophages and discussion. JPM provided laboratory equipment, reagents and discussion. HRS oversaw the study, provided the MSU crystals and initial instruction in the use of the air pouch model, and edited the manuscript. All authors read and approved the final manuscript.
